# Advanced Organotypic In Vitro Model Systems for Host–Microbial Coculture

**DOI:** 10.1007/s13206-023-00103-5

**Published:** 2023-05-22

**Authors:** Raehyun Kim

**Affiliations:** Department of Biological and Chemical Engineering, Hongik University, Sejong, Republic of Korea

**Keywords:** Organotypic model systems, Microphysiological systems, Bacterial coculture, Commensal bacteria

## Abstract

In vitro model systems have been advanced to recapitulate important physiological features of the target organ in vivo more closely than the conventional cell line cultures on a petri dish. The advanced organotypic model systems can be used as a complementary or alternative tool for various testing and screening. Numerous data from germ-free animal studies and genome sequencings of clinical samples indicate that human microbiota is an essential part of the human body, but current in vitro model systems rarely include them, which can be one of the reasons for the discrepancy in the tissue phenotypes and outcome of therapeutic intervention between in vivo and in vitro tissues. A coculture model system with appropriate microbes and host cells may have great potential to bridge the gap between the in vitro model and the in vivo counterpart. However, successfully integrating two species in one system introduces new variables to consider and poses new challenges to overcome. This review aims to provide perspectives on the important factors that should be considered for developing organotypic bacterial coculture models. Recent advances in various organotypic bacterial coculture models are highlighted. Finally, challenges and opportunities in developing organotypic microbial coculture models are also discussed.

## Introduction

A healthy human body hosts hundreds of trillion microbes that make up about 1.3 times more bacterial cells than human cells [1]. Since the germ theory of disease was established and widely accepted, microbes have been associated mainly with pathogenesis for a long time, while the existence and roles of commensal microbes have been largely neglected. However, in the last 2 decades, myriad research has revealed that commensal microbes dynamically interact with the host and exogenous pathogens, and these interactions are associated with a broad range of health-related conditions of the host, including immune system development and training, [2, 3] physiological processes such as pregnancy and aging [4, 5], health and disease states [6–8], and efficacy of therapeutic interventions [9, 10]. Understanding host–microbial interactions, both pathogens and commensals, can offer new ways of diagnostic, therapeutic and preventative strategies of diseases, and insight of maintaining and promoting health.

Animal models have been the main experimental tools for investigating host–microbial interactions in the in vivo setting. However, the relatively high cost, low reliability, and ethical issues significantly limit the accessibility of animal models. Also, importantly, animal models cannot fully mimic the host–microbial interactions occurring in the human, body because various host factors that regulate microbiota, such as anatomy, physiology, genetics, diet, and life cycles, of model animals are different from those of humans. These interspecies differences likely cause disparities between pre-clinical animal studies and the clinical results with the human subjects [11, 12]. An in vitro microbial coculture model with human cells can be a complementary and alternative model to animal models. Generally, in vitro model systems are less costly and easier to run experiments on than animal models. In some cases, in vitro model systems are biologically more suitable than animal models. For example, some pathological phenotypes, such as pulmonary surfactant-free phenotype, cannot be generated in model animals due to their lethality. In contrast, an in vitro model system consisting of a subset of cells can recapitulate the pathobiology [13]. Moreover, certain infections that occur in humans cannot be fully recapitulated in animal models. Enterohemorrhagic *Escherichia coli* (EHEC) infection in mice requires a reduction of commensal bacteria prior and much higher bacterial loading compared to human hosts (10^7^ vs. 10^2^ microbes) [14]. A well-designed in vitro coculture model system with human cells can offer an efficient way to study such pathogenic infections through directly observing and measuring physical and biochemical interactions between the microbes and the host cells with easier manipulation of variables.

Recently, various innovative in vitro model systems successfully recapitulated organ-specific biological and physiological features of in vivo tissues. Still, most of these systems are missing essential partners of the human body, microbes. The tremendous amount of research on the human microbiome suggests that introducing bacteria to the organotypic model systems may fill the gap between the in vivo and in vitro tissue and offer a comprehensive view of how the target organ works. This review aims to provide perspectives on the important factors to consider in coculturing bacteria with human cells and introduce recent developments of various organotypic in vitro coculture model systems. It was intended to focus on commensal bacterial coculture models, but due to the paucity of such studies, pathogenic bacterial coculture studies with significant importance are also discussed. And finally, challenges and opportunities unique to bacterial cocultures in vitro systems are discussed.

## Brief Introduction to the Human Microbiota and Human Epithelium In Vivo

Microbes and epithelial tissue are the essential components of an in vitro bacterial coculture system. In a healthy human body, commensal or pathogenic microbes are usually first checked and handled by the epithelial tissues. Microbes influence the host through direct contact (in some cases as pathogen infection) or secreting soluble substances that are absorbed, transported by, or pass through the epithelial cells. These interactions can initiate signaling events or the recruitment of other cell types. Here, I briefly summarize basic knowledge of human microbiota and the epithelial tissues in vivo that should be considered in preparing in vitro model systems.

### Human Microbiota

The microbiome of a healthy person is estimated to contain 100 times more genes than human genes (~ 2,000,000 vs. ~ 20,000) [15]. Among these diverse microbes residing in the human body, including viruses, fungi, and archaea, bacterial compositions are the best characterized through national and international projects such as the Human Microbiome Project (HMP), funded by the National Institutes of Health of the US, and the MetaHit project run by thirteen teams from eight countries. These studies generated a lot of valuable data and information, including reference catalogs of the human microbiomes, metagenomic, and 16S metagenomic sequence databases from a large population of donors, protocols for samplings, data processing, and data analyses of the microbiome data [16–18]. These studies revealed that various host factors, including genetics, diets, lifestyles, and medical interventions, can alter microbiome compositions [19]. As a result, each healthy individual has unique gut microbiota that responds dynamically to sudden changes of the host factors but also maintains the uniqueness and robustness of the microbiome over a long time. The vast individual and temporal variability of the human microbiome make it challenging to define a “healthy microbiome” [19].

In addition, numerous factors specific to the location in a human body, such as topology, anatomy, physiology, frequency of washout, and replenishment of nutrients and resources, shape the microbiota with bacterial loads, compositions, diversities, and stability that are unique to the location. The gastrointestinal tract hosts most of the human microbiota, mainly colonized in the colon (large intestine, ~ 10^14^), overwhelmingly outnumbering the bacterial loads of other digestive organs (small intestine, ~ 10^11^, stomach ~ 10^7^) or other sites of the body [20]. Oral and skin bacterial populations are estimated to be ~ 10^12^ and ~ 10^11^, respectively [20], and about 10^11^ bacteria are estimated to reside in the female genital tract [21]. Estimated biomass in the lung is 10^3^ to 10^5^ per gram of tissue, making total of 10^6^ to 10.^8^ bacteria in an adult lung [22]. The implication of bacterial diversity is also organ-specific. The gut microbiome exhibits the highest diversity, and the degree of diversity is correlated with the healthy condition of the host. In contrast, the microbiomes of the female reproductive tract are relatively similar across individuals enough to be used for classifying different pathological states, and a higher diversity of vaginal microbiota is associated with compromised barrier functions and a higher risk of adverse events such as vaginal infections [23]. The stability of the microbiome also differs by the site. Gut microbiomes in healthy adults are relatively more stable than skin and vaginal microbiomes [24].

The human microbiome data revealed that the compositions of commensal bacteria are closely related with the health condition of the host. In healthy conditions, a well-balanced state between the host and the commensal microbes, eubiosis, is maintained by immune systems that have microbes under control and commensal microbes that prevent pathogen colonization. Microbial dysbiosis is associated with diseased conditions of the host. Patients with various non-infectious diseases exhibit microbiomes distinct from healthy subjects, suggesting that disease-associated microbiomes can be biomarkers for disease [25]. It has been actively investigated whether microbial dysbiosis is an indication of a disease condition or can cause illness. Many studies with (germ-free) animal models suggest that modulating microbial composition can be an effective therapeutic intervention strategy. A comprehensive understanding of host–microbial interactions, beyond cataloging the microbial compositions, is urgently necessary to utilize the microbiota for enhancing health.

### Human Epithelial Cells and Their Communications with Immune Cells

The epithelia are sheet-like cell layers that form the linings of the internal and external organs. Usually, epithelia are continuously regenerated in a healthy, homeostatic state to replace damaged or dead cells by the harsh environment they face. Also, epithelial tissues are equipped with defense mechanisms such as the generation of mucus or glycosaminoglycans (GAGs) and the secretion of antimicrobial peptides. Epithelial tissues usually adhere to the base membrane and communicate with other cell types in proximity, such as stromal cells, immune cells, nerve cells, endothelial cells, and circulating blood cells.

Epithelial cells found in the human body have one of these distinct shapes, namely squamous (thin and flat), cuboidal (cubic-like), and columnar (tall) epithelial cells. Epithelial cells are polarized into apical (lumen or exterior facing) and basolateral domains (in contact with the base membrane), which is critical for functioning properly. Epithelial cells organize themselves to form epithelial tissues or epithelium [26]. Epithelial tissue organizations can be classified into simple, stratified, and pseudostratified epithelia. Simple epithelia consist of a single layer of these epithelial cells. They usually involve transport such as absorption and excretion and secrete hormones, enzymes, and protective substances, including mucus and antimicrobial peptides. The gastrointestinal tract (stomach, small intestine, and colon), various glands, including liver and mammary salivary glands, and alveoli contain simple epithelium consisting of columnar, cuboidal, and squamous epithelial cells, respectively. Stratified epithelium, found in the skin, esophagus, and vagina, comprises two or more layers of epithelial cells. One side of the epithelium is in contact with the base membrane, and the other faces the outer environment. The pseudostratified epithelium is a single layer of epithelial cells that appears to be stratified due to the different heights of the nuclei in different cell types. Part of the respiratory tract is lined with ciliated pseudostratified epithelium. For developing organotypic in vitro model systems with epithelial cells, it is crucial to reproduce the correct epithelial shapes and organization, as well as the cell behaviors of the target organ.

Epithelial tissues communicate with the immune systems to deal with microbes. When pathogens compromise epithelium, innate immune system components are rapidly recruited, in part through the epithelial cytokines, to clear out the risk factors in the early stage of infection and prevent the pathogens from spreading through circulation. Adaptive immune systems provide another line of protection specific to previously encountered pathogens. Meanwhile, in healthy epithelial tissues, symbiotic or commensal microbes escape from the clearing process of the host immune system. In the digestive tract, discerning commensals from pathogens involves continuous sampling and monitoring of microbiota by the host immune systems. For example, antigen-presenting immune cells (e.g., dendritic cells or macrophages) migrate or stretch their projections through endothelial or epithelial cells to sample microbes in the mucosa or lumen. Also, a subset of epithelial cells, such as goblet cells or M-cells in the oral cavity or small intestine, engulf microbes and present them to the immune systems [27]. Actually, commensal microbes are critical components for the proper development of host immune systems. Germ-free mice exhibited impaired immune cell differentiations and reduced immunoglobulin production, clearly indicating the crucial roles of microbes in proper immune system development [3, 28]. The interaction mechanisms of the immune system, the epithelium, and the microbiota are actively investigated and expected to provide a deeper and broader understanding of health and disease conditions.

## Considerations for Developing an In Vitro Coculture Model Systems

**Fig. 1:**
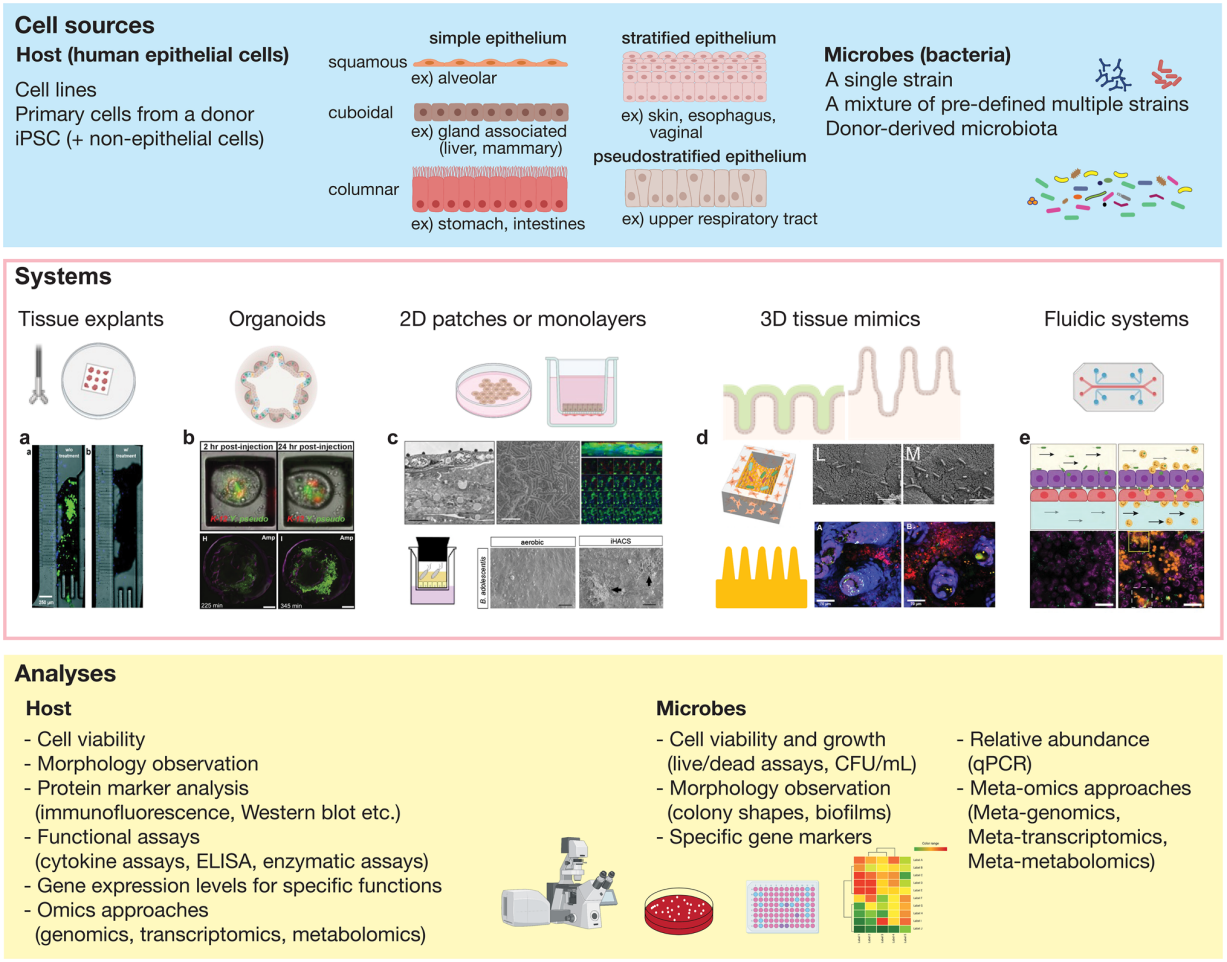
Considerations for building an in vitro coculture model system. (Top) the cell sources of host epithelial (left) and bacterial (right) cells used for developing an organotypic coculture model. Host epithelial cell types and epithelial organizations are shown in the middle. (Middle) Various in vitro coculture systems have been developed, including tissue explants, organoids, two-dimensional monolayers, micro-structured tissue mimics, or microfluidic devices. **a** A tissue explant with bacterial infection was cultured in a microfluidic device. Antibiotic treatment removed the GFP-labeled bacteria (green) from the tissue explants (black). Reproduced with permission from [88]. **b** Bacterial coculture in organoid cultures. Top: human intestinal organoids microinjected with DsRED-expressing *E coli* (red) resistant to tetracycline and GFP-expressing *Yersinia pseudotuberculosis* (green). Reproduced with permission from [35]. Bottom: mouse bladder organoids microinjected with uropathogenic *E. coli* (UPEC). UPEC expanded in the organoid wall even in the presence of an antibiotic (ampicillin). Reproduced with permission from [114]. **c** Examples of two-dimensional coculture model systems. Top: from left to right, transmission electron microscopic image, scanning electron microscopic image, and fluorescent microscopic image of vaginal commensal bacteria cocultured with the immortalized vaginal epithelial cells. Reproduced with permission from [29]. Bottom: a commensal gut bacterial species (*Bifidobacterium adolescentis*) cocultured with the human primary intestinal epithelial cells. To coculture the anaerobic gut bacterial strain, anaerobic air was introduced into the apical side of the device depicted on the left. Reproduced with permission from [78]. **d** Three-dimensional tissue models with bacterial coculture. Top: an intestinal model system fabricated with silk scaffold cocultured with human gut microbiota. Reproduced with permission from [30]. Bottom: a three-dimensional intestinal model system with intestinal epithelial cells (blue) on a hydrogel scaffold cocultured with probiotic strain (red) and pathogen (green). Reproduced with permission from [38]. **e** Microfluidic system with bacterial coculture. Human bladder epithelial cells (magenta), endothelial cells (not shown), and neutrophils (amber) in a bladder-on-chip device cocultured with UPEC (green). Reproduced with permission from [42]. The image was created using BioRender.com

In vitro model systems have been increasingly advanced to represent the human body more closely. Incorporating bacteria into an in vitro system is another crucial step in creating a more comprehensive and complete model of our body, considering the significance of microbes in host health conditions implicated in animal studies and clinical data. Bacterial coculture systems can provide platforms to delineate the host–microbial interactions in detail and potentially offer more “in vivo human-like” pre-clinical test platforms for drug development. To utilize bacterial coculture systems efficiently, it is essential to verify whether it accurately mimics the important events occurring in vivo and whether any experimental conditions artificially alter the outcome. Here, essential elements in building an organotypic bacterial coculture model system and characterization methods are discussed and summarized in Fig. 1.

### Coculture Formats

Various human epithelial cell culture formats have also been used for organotypic bacterial cocultures. The simplest way to culture epithelial cells is growing them on two-dimensional rigid surfaces, such as Petri dishes, well plates, flasks, or glass. This traditional way to culture mammalian epithelial cells is still commonly used to expand and maintain cells. However, cells on rigid surfaces behave very differently from the cells in vivo in the same organ, because the environments that the cells are exposed to, such as stiffness or permeability, are very different. On a stiff surface, columnar epithelial cells become squamous and lose polarization. Also, this configuration has a single compartment for liquid, which makes it impossible to distinguish apical and basolateral compartments that correspond to the lumen or the exterior and circulation, respectively, in vivo. In this simple two-dimensional model system, bacterial coculture is conducted by simply inoculating bacteria in the medium. While bacterial coculture is not impossible, it is unlikely that the cell behaviors in this format represent the in vivo tissue, because the biochemical and physiological environment is far from those in vivo.

Porous membrane-assisted cell culture is one of the most frequently used and readily accessible methods for in vitro epithelial model systems. Cell culture inserts (e.g., Transwell, MilliCell, etc.) are commercially available cell culture devices with a porous membrane. They are used as a companion to a standard well plate, and different pore sizes and membrane materials are available. Porous membranes, usually made of thin plastic films, such as polyethylene terephthalate (PET), polycarbonate (PC), or polytetrafluoroethylene (PTFE), offer physical support for cells to grow while enabling biochemical transport through the micro-sized pores. This configuration effectively separates apical and basolateral compartments, which correspond to the cell culture insert and the well plate, with unlimited and independent access for sampling or adding test compounds. Also, it enables culturing the cells in the air–liquid interface by removing liquid from the apical side, mimicking the in vivo environments of the tissues exposed to a gas phase, such as the skin, airway, or vaginal epithelium. Epithelial cells plated on porous membranes are usually polarized properly upon confluence and exhibit directional transport. Using a porous membrane allows the incorporation of various cells into the epithelial cell culture for multicellular tissue models. Adherent cells, such as stromal cells or endothelial cells, are typically attached on the other side of the epithelial cells attached. Non-adhering cells or bacteria can be added into the appropriate compartment. Porous membrane-assisted culture is often adopted in bioengineered systems, such as microfluidic devices. For bacterial cocultures in this format, bacteria are inoculated into the apical side of the epithelial cultures, reflecting the in vivo environment. Typically, epithelial cells are cultured on PET or PC membranes with 0.4 μm pores which are smaller than typical sizes of bacteria. In this case, any bacteria found in the basolateral compartment are likely unintended contamination that occurs during sample preparation or manipulation, rather than the target bacteria passing through the pores from the apical side to the basolateral side during cocultures.

Organoid culture is an increasingly popular cell culture format for its capability to preserve genotypes and phenotypes of donors in primary tissues and induced pluripotent stem cells (IPSCs). Organoids are usually prepared by embedding the primary cells or IPSCs in a hydrogel. These epithelial organoids typically form closed hollow structures. This 3D structure embedded in hydrogel significantly limits access to the luminal side of the organoids and imposes challenges for various analyses, including solution-based assays and microscopic imaging. Also, in this configuration, the density or size of the organoids is hardly controlled. However, new organoid preparation approaches have been developed to address these issues, such as inside-out organoids or suspension culture of organoids for easier and faster organoid production. [31, 32] Other innovative organoid technologies for recapitulating the complexity of the in vivo tissue with higher controllability can be found elsewhere [33]. For coculturing bacteria in the apical side of the organoids, bacteria need to be microinjected into the lumen of organoids. Typical microinjection involves the microscopic detection of individual organoids and the positioning of a microneedle for inoculating bacteria into the organoids, which is time-consuming and has low throughput. An automated setup can improve the throughput of microinjection, but the setup is not yet readily available [34, 35]. Alternatively, the use of “inside-out” or “apical-out” organoids allows inoculating bacteria directly into the culture medium. “Inside-out” or “apical-out” organoids are prepared by dissociating the ECM of the usual organoids culture which results in inverting the polarity of the organoids [31]. In this case, bacteria can be directly added into the culture medium to interact with the apical side of the epithelium [31].

Bioengineered organotypic model systems, also called microphysiological systems (MPS), employ diverse engineering strategies to recapitulate key physiological features of target organs. One of the crucial features of the tissue is its three-dimensional microstructure. In vivo tissues are spatially organized with various cells, and this spatial organization is critical in tissue homeostasis and functions [36, 37]. Various fabrication methods, including microstamping or 3D printing, have been used to create 3D micro-structured scaffolds with the same microscale dimensions of the in vivo tissues, such as the crypt (small and large intestine) [37], villi (small intestine) [38], or alveoli [39]. Bacterial coculture in micro-structured in vitro models can offer platforms to recreate and observe the biogeography of host–bacterial interactions, such as spatial distributions of the commensal or pathogenic bacteria and their effects on the host cell phenotypes or immune responses.

Organ-on-chip is one of the well-recognized MPSs, which consists of engineered or native tissues cultured inside microfluidic chips [40]. Most organ-on-chip models have two channels separated by a porous membrane. Epithelial cells are seeded on the top-facing side of the porous membrane, and optionally, vascular endothelial or stromal cells can be introduced on the other side. Designing microfluidic systems requires significant expertise in microfabrication and manipulating liquids in tubing and pumps, which may limit access to broader users. Dynamic features that conventional culture methods cannot mimic, such as continuous blood flow, cyclic stretching, or filling and emptying of the bladder, have been recapitulated in organ-on-chip models [41, 42]. Bacterial cocultures in microfluidic systems are conducted by flowing microbes in the apical channels. One significant advantage of using fluidic systems for bacterial coculture is that continuous or pulsatile perfusion can remove excessive microbes and microbe-derived toxic materials and replenish fresh medium, contributing to a long-term coculture capability.

Target specifications in designing and developing organotypic bacterial coculture models differ by the physiological characteristics of target organs and the biological requirements of target bacteria. Periodic stretching may play significant roles for lung models but not as much for skin models. Most of the commensal bacteria in the intestine are obligate anaerobes, as described later. Therefore, for intestinal commensal bacterial coculture models, oxygen conditions must be met for both host cells (which require oxygen) and bacteria (that cannot survive in the presence of oxygen) for successful coculture. Some of those requirements pose various engineering challenges and invite innovative approaches to designing a system. In addition to mimicking the in vivo organs, a systematic design to prevent contamination or cross-contamination is highly for the microbial coculture model.

### Host Cell Types and Sources

Generally, current standard pre-clinical in vitro experiments utilize human cell lines derived from cancer or immortalized. Despite the aberrant and unstable mutations and phenotypes that are distant from the in vivo cells [43, 44], immortalized cell lines are easy to obtain from cell depositories or commercial sources and maintain. Also, cell lines provide consistency across the studies to a certain extent once authenticated.

Recent developments in primary stem cell culture methods enable long-term culture and preservation of non-transformed primary cells obtained from biopsy, surgical samples, or cadaveric donors. Culturing non-transformed primary cells can preserve the genomic information of the donor and mimic the in vivo phenotypes more accurately than transformed cell lines [43]. The primary epithelial cells from donors contain stem cells for continuous regeneration or exhibit the plasticity to convert themselves to proliferative cells in the right conditions [45, 46]. Tissue-specific medium compositions for growing and differentiating the primary epithelial cell culture have been developed as signaling pathways involved in proliferation and differentiation were revealed [47–49].

IPSCs are another important cell source for building an organotypic model system. IPSCs are reprogrammed cells to a pluripotent state, usually from somatic cells obtained by a less invasive procedure (e.g., skin biopsy), and differentiate into various tissue-specific cell types expressing in vivo-like phenotypes. It takes long (~ several weeks) to fully differentiate IPSCs into a tissue-specific mature epithelium than primary epithelial cells, with a series of medium changes with different medium compositions. Still, it can differentiate into various organotypic cells with established protocols for target tissues. In most cases, tissue-specific epithelium differentiated from IPSCs is accompanied by the stromal cells underneath.

An appropriate extracellular matrix (ECM) is required for cells to express proper phenotypes. Some cell lines do not require external ECM, since they can secrete ECM themselves, but still, external ECM accelerates cell attachment and growth. For primary epithelial cells, external ECM is necessary for cell attachment and proliferation. Typically, primary cells or IPSCs are embedded (e.g., organoid culture) in or laid over on hydrogels (2D or 3D monolayer or patches) for long-term culture and preservation [47, 50, 51]. A thin coating of ECM on the substrate is often used and sufficient for functional assays. Native or synthetic hydrogels such as Matrigel, collagen, or polyethylene glycol (PEG) offer physical and mechanical properties supporting cell adhesion, growth, and expansion.

Immune cells are increasingly incorporated into organotypic in vitro model systems to capture bacteria–epithelial–immune cell interactions. Cell lines with established differentiation protocols or primary immune cells isolated from blood, including neutrophils, macrophages, monocytes, dendritic cells, natural killer cells, T cells, or peripheral blood mononuclear cells (PBMCs), have been introduced to organotypic model systems [52–58]. Integrating innate immune cells can elicit organ-specific immune responses to commensal bacteria or pathogen infections. Profile changes of cytokines or inflammation markers by microbial challenges or probiotic intervention are assessed by solution or cell-based assays, such as ELISA, cytokine arrays, or gene expression analyses. Immune cell defense mechanisms in action, including transepithelial projections, transepithelial and transendothelial migration (if endothelial cells exist in the model), and the engulfment of immune cells in response to bacteria and cytokine profile changes, can be observed by microscopy.

The incredible ability of a healthy human body to cope with microbes suggests that expressing healthy in vivo tissue-like cell phenotypes, such as producing mucus layer or respiratory surfactants, likely yield a more successful coculture. In this sense, primary cells or IPSCs are more suitable than transformed cell lines for bacterial coculture despite higher expenses, since they can express in vivo-like genotypes and phenotypes that the transformed cell lines have lost. Meanwhile, the missing phenotypes of the human cells in vitro may be caused, at least in part, by the absence of the bacteria. Bacterial coculture models, with proper controls, can offer ways to answer the questions on bacteria-driven host cell phenotypes and elicit the roles of bacteria in human tissue development.

### Bacteria

Bacteria can be obtained from non-profit depositories [e.g., American Type Culture Collection (ATCC), German Collection of Microorganisms and Cell Cultures (DSMZ), Japan Collection of Microorganisms (JCM), Korean Collection for Type Culture (KCTC), and National Collection of Type Culture (NCTC, UK)], or for-profit commercial sources. A type strain of a bacterium is defined as descendants of the original isolates used for designating the species and subspecies of the bacteria. Type strains are valuable for comparative and reference work worldwide, similar to human cell lines [59–61].

Clinical isolates from donors obtained at medical facilities have been cultured and cocultured with human cells. The bacteria present in the clinical samples can be identified through 16S rRNA or whole genome sequencing. A considerable number of gut bacteria discovered by genomic sequencing are still not culturable due to difficulties in optimizing the culture conditions, but recently, the culturability of gut bacteria has been improved significantly by screening various supplements or developing universal media that can elicit the highest number of bacterial species from donor-derived gut microbes [62, 63]. Clinical samples with the microbial community are often subjected to coculture without expanding to avoid artificial composition changes.

Usually, members of human microbiota in an optimized culture condition grow faster and require fewer maintenance and expensive components than human host cells. However, some fastidious bacteria may need special equipment or supplements. For instance, most gut bacteria are obligate anaerobic bacteria that cannot survive when exposed to oxygen. Thus, culturing and manipulating them require an anaerobic environment, such as an anaerobic chamber or a closed vessel filled with an anaerobic gas (e.g., GasPak).

### Conducting Coculture Experiments and Analyses

Bacterial coculture experiments are performed by inoculating bacteria into human epithelial cells in an antibiotic-free medium. Broadly speaking, two strategies have been employed. One is to coculture the entire population of bacteria for the entire coculture period without changing the culture medium, and the other is to coculture only a subset of the bacteria that remains bound after a short initial incubation (typically 30 min to 2 h) and subsequent washout. The aim of the study, types of coculture systems (e.g., static or dynamic system), and tolerance levels of the host cells are taken into account to plan bacterial coculture experiments. Proper negative controls of both host and bacterial cells, such as human cells without bacterial exposure and bacteria without human cell exposure, are necessary and useful for figuring out the influence of the bacteria on the human cells or vice versa and for possible troubleshooting.

Evaluation and analysis methods of the bacterial coculture experiments may vary according to the aims of the study. Importantly, whether both host cells and bacteria are sufficiently viable and functional during the coculture should be verified, particularly in commensal bacterial coculture. For viability assays, cell-permeable and impermeable fluorescent nucleic markers are often used on the host and bacterial cells with microscopy. The number of viable bacteria can also be quantified in the unit of colony forming unit (CFU) by inoculating the bacteria on agar plates and counting the colonies (since only viable bacteria can make colonies). Optical density or absorbance measurement (usually at 600 nm) also estimates the number of bacteria in the solution, but it does not distinguish live bacteria from dead ones. For simple epithelium, barrier properties are characterized by immunofluorescence for tight junction markers, permeability assays, and transepithelial electrical resistance (TEER) measurements. For differentiated stratified epithelium, the existence of multiple layers or a single layer of cells covered with a keratin layer should be verified. Immunofluorescences and various functional assays can further characterize the host cells. Once the models are confirmed to be adequate for presenting the host–microbial interactions, the effects of the bacteria on the host cells or vice versa can be characterized using various assays and measurements. Table 1 summarizes assays and analyses commonly performed in bacterial coculture experiments.

**Table 1:** Commonly used characterization methods for bacterial coculture studies

	Functions, phenotypes	Assays, tests
Human (host) epithelial cells	Viability or cell death	Live/dead assay MTT, MTS, and similar assays (Cell Titer Glo) LDH assays
	Epithelial integrity, barrier functions	Transepithelial electrical resistance (TEER) Permeability assays
	Morphological changes (columnar or squamous, microvilli etc.)	Fluorescence microscopy Electron microscopy
	Protein expressions (differentiation markers, functional proteins such as transporters or enzymes, protein secretions)	Immunofluorescence Histological staining Proteomics analysis (mass spectrometry) Functional assays for specific proteins ELISA Western blot
	Gene expressions	RT-qPCR mRNA sequencing
	Metabolisms (bile acids, fatty acids, etc.)	GC/MS or LC/MS Metabolomics analysis
	Immunological responses (pro-inflammatory cytokines: TNF-α, IL-1, IL-6. IL-8, IL-17, IFN-γ, MCP-1, G-CSF, etc.; anti-inflammatory cytokines: IL-4, IL-10, IL-13, IL-22, TGF-β, etc.)	Cytokine screening panels ELISA
Bacteria	Presence and morphology	Fluorescence microscopy Electron microscopy In situ hybridization
	Host cell binding	Counting from images CFU measurement of harvested bacteria from the host cells
	Viability or cell death	Live/dead assays (cell-based, microscopic) CFU estimation
	Bacterial growth	Comparison of the number of viable bacteria by counting colony forming unit (CFU) from *t* = 0 and the end of the coculture Optical density difference qPCR
	Bacterial metabolites	GC/MS or LC/MS
	Bacterial toxins	ELISA
	Compositional changes (in mixed population of multiple bacteria)	qPCR Sequencing

## Current Developments of Organotypic Coculture Model Systems

This section discusses organ-specific considerations for developing in vitro bacterial coculture systems. Additionally, recent studies on bacterial coculture are highlighted with a focus on commensal bacteria and noteworthy studies on pathogenic coculture (Table 2).

**Table 2:** Examples of organotypic microphysiological systems with bacterial cocultures

Organ	Culture system	Host cells	Bacteria	Key findings	References
Intestine	3D micro-structured hydrogel	Human colon cancer cell line	*Lactobacillus gasseri* 33323 and *E. coli Nissle* 1917 (probiotics) + *Salmonella typhimurium* 14038, *Pseudomonas aeruginosa* 15692 (pathogen)	Improved host cell survival against pathogenic infection by probiotic strains Different spatial distribution of bacterial strains in the 3D crypt-villi axis	[38]
	Porous membrane-assisted	Human epithelial cell lines + monocytes/macrophage cell line embedded in collagen gel	*Escherichia coli*-TOP10 GFP (commensal)	Immune competent system Stable coculture with a simple commensal bacterium only in the presence of macrophage Decreased damages of the epithelium and altered cytokine profiles by excretory/secretory products (ESPs) of the helminth in the bacterial-epithelial-immune cell model	[68]
	Microfluidics: HuMix	Human colon cancer cell line Patient-derived colorectal cancer cell line	*Bacteroides caccae* (commensal) + *Lactobacillus rhamnosus GG* (*LGG*, probiotic) *Fusobacterium nucleatum* (a colorectal cancer-related commensal)	Physiological oxygen gradient In a microfluidic model with three channels separated by a nanoporous membrane and a micro-sized porous membrane Distinct transcriptional responses by coculturing *B. caccae* and *LGG* than coculturing *LGG* alone Alteration of bacteria-driven gene expression pattern by different oxygen environments (with and without oxygen gradient) Used to screen cancer-related metabolites	[69, 70]
	Microfluidics : intestine chip	Human primary ileal epithelial cells + primary endothelial cells	*Bacteroides fragilis* (*B. fragilis*, commensal) A complex community of gut bacteria collected from human feces and maintained in gnotobiotic mice (commensal)	Physiological oxygen gradient with apical and basolateral flows Increased intestinal barrier function and physiological level of microbial diversity maintained by establishing the oxygen gradient The peristaltic movement not necessary for successful coculture	[72]
	Microfluidics : anoxic–oxic interface (AOI) on-chip	Human colon epithelial cell line	*Bifidobacterium adolescentis* (*B. adolescentis,* commensal), *Anaerobutyricum hallii* (*A. hallii,* commensal)	Physiological oxygen gradient with apical and basolateral flows High viability of both bacteria and the epithelial cells	[74]
	Meso-fluidics: gut-microbiome physiomimetic platform (GuMI), continuous apical flow and recirculating basolateral flow	Organoid-derived primary human colon epithelial cells	*Faecalibacterium prausnitzii* (*F. prausnitzii, commensal*)	Physiological oxygen gradient with apical flow and recirculating basolateral flow Facilitated differentiation by apical and basolateral flows than static culture conditions Increased hypoxia-inducible factor 1-α (HIF-1α) gene and related effector gene expressions by the oxygen gradient compared to a conventional aerobic condition Altered gene expression patterns related to inflammation and Toll-like receptor (TLR) signaling toward anti-inflammation by coculturing *F. Prausnitzii*	[75]
	Microfluidics: gut microbial- epithelial cell coculture system (GMEC), a pumpless system with gravity-driven flow	Human colon cancer cell line	*Lactobacillus plantarum* (*L. plantarum*, probiotic)*,* GFP-*E. coli* (commensal)	Physiological oxygen gradient Enhanced barrier function of *L. plantarum* in various forms (live, crushed, lyophilized) in aerobic condition With an optimized flow rate, successful coculture of *E. coli* without losing viability	[76]
	Porous membrane-assisted	Human primary colon epithelial cells	*LGG* (probiotic) *Clostridioides difficile* (commensal) *LGG* (probiotic), *B. adolescentis* (commensal)*,* *A. hallii,* (commensal/probiotic)	Physiological oxygen gradient Improved tolerance to *Clostridioides difficile* infection in the oxygen gradient condition compared to an anaerobic condition A donor-invariant anti-inflammatory effect of a butyrate-producing bacteria *A. Hallii* while donor-dependent tolerance to *LGG*	[77, 79]
	Porous membrane-assisted: intestinal hemi-anaerobic coculture system (iHACS)	Human primary epithelial cells	*B. adolescentis, B. fragilis*, *Clostridium butyricum, Akkermansia municiphila *(all commensals, probiotic)	Physiological oxygen gradient No external nutrient necessary for coculturing a mucus-degrading *Akkermansia municiphila*	[78]
	Organoids	Human intestinal organoids Mice colon organoids in high-throughput microinjection platform	Genotoxic *E. coli* (pathogen) GFP-expressing *E. coli* NC101, *E. coli* K12*-*DsRED, *Yersinia pseudotuberculosis–*GFP, Bacterial community from human stool, *Bifidobacterium adolescentis* (commensal)	Microinjection of microbes Distinct mutational signatures induced by 5 months of genotoxic *E. coli* injection that are overlapped with a subset of human cancer genomes. Detailed protocol described in [34] GFP-expressed bacteria detected in the organoids after microinjection 96 h of cultivation of human gut microbial community in the high-throughput microinjection platform	[﻿^34^, ^35^, ^80^]
Skins	Porous membrane-assisted	Commercial full-thickness 3D skin model cultured in ALI (human normal epidermal keratinocytes + dermal fibroblasts)	*Micrococcus luteus*, *Pseudomonas oleovorans* separately, together (commensal from healthy skin)	Species-dependent alterations of secreted cytokines, antimicrobial peptides, and growth factors Influence of one strain (*P. oleovoran*s) dominating over the other (*M. luteus*) upon simultaneous coculturing with two strains	[85]
	Porous membrane-assisted	Commercial full-thickness skin tissue model consisting of epidermal and dermal layers grown in ALI	Eight different bacterial strains and a mixed community	Distinct transcriptional changes induced by mixed community of bacteria from individual bacterial treatment The most prominent phenotype changes by the mixed community than individual strains (a decreased thickness of the nucleated epidermal layer and proliferative cell populations) Significant increases in the expression of skin barrier function-related genes (loricrin and filaggrin) only in the mixed community treated samples	[86]
	Porous membrane-assisted	A double-layer model of keratinocytes over fibroblast embedded in fibrin hydrogel	*Burkholderia thailandensis, *a surrogate model strain for *Burkholderia pseudomallei*, a pathogen for melioidosis	Delayed wound healing, triggered inflammasome, and ejection of the keratinocytes by the infection	[87]
	Microfluidics	Full-thickness skin explant + primary neutrophils in a microfluidic system	*Staphylococcus aureus*	Migration of neutrophils toward the infected tissues and not the uninfected tissues Reduced neutrophil migration by antibiotic treatment	[88]
Female reproductive tract	Monolayer on tissue culture plate, porous membrane-assisted	Immortalized epithelial cells from different regions on well plates and Transwell in ALI	Commensal BV-associated bacteria	More sensitive and robust immune responses of the cells in the upper female genital tract Increased secretions of pro-inflammatory cytokines (IL-8, Gro-alpha (CXCL1), and antimicrobial peptide hBD2 by BV-associated bacteria and *Lactobacillus vaginalis,* than the commensal bacteria	[95]
	Monolayer on tissue culture plate, porous membrane	Monolayers of immortalized endocervical, ectocervical, and vaginal epithelial cells, Primary polarized 3D ectocervical tissue model (VEC-100)	*Prevotella bivia* (*P. bivia,* BV-associated), *Atopobium vaginae* (*A. vaginae,* BV-associated) *Lactobacillus crispatus* (*L. crispatus,* commensal)*, Lactobacillus acidophilus* (commensals)	Anaerobic coculture Higher secretion of IL-8 and NF-κB in coculture with BV-associated bacteria than in coculture with commensal strains Microbicidal compound cellulose sulfate-induced increase in expression of IL-8 and secretory leukocyte protease inhibitor in a dose-dependent manner	[96]
	3D aggregates in a rotating wall vessel reactor	3D cell aggregates of immortalized primary cells on collagen-coated dextran beads in a rotating vessel reactor Anaerobically	*Lactobacillus iners* (commensal), *L. crispatus* (commensal) *P. bivia* (BV-associated), *A. vaginae* (BV-associated)	Increase in the gene expression of some pro-inflammatory cytokines, antimicrobial peptide defensins, and protein secretions of cytokines, including IL-6, IL-8, TNF-α, and IL-1β, by a BV-associated bacterial strain	[97]
	Microfluidics	Primary vaginal epithelial cells, uterine fibroblast cells	A strain or multi-strain consortia of *L. crispatus* (commensal)	Altered gene expression levels of estrogen receptor 1 (ESR1), progesterone receptor (PGR), and claudin 17 (CLDN17) by β-estradiol 4 days of successful coculture with a strain or multi-strain consortia of *L. crispatus* Suppressed pro-inflammatory cytokine secretions (IL-8, IL-6, IL-1a, IL-1β, and IP-10) by commensal bacterial coculture BV-associated bacterial coculture induced increase in pH and the pro-inflammatory cytokines, while no lactate detected, compared to the control without bacterial exposure	[98]
Respiratory	Porous membrane-assisted	Airway epithelial cell lines (Calu-3) + monocyte/macrophage cell line in ALI	A nasal bacterial community (commensal), *Lactobacillus sakei* (probiotic), *Staphylococcus aureus* (pathogen)	Successful coculture Alteration of microbiome compositions and diversity by adding THP-1 macrophage-like cells	[101]
	Microfluidic	Epithelial + endothelial + neutrophils in Lung on a chip	GFP-*E. coli* (model bacterial)	Transendothelial and transepithelial migration of the neutrophils and the phagocytosis of GFP-*E. coli* under inflammatory stimulation with TNF-α Increased reactive oxygen species in the cells by nanoparticles upon cyclic strains	[41]
	Microfluidics	Primary alveolar epithelial cells	*Mycobacterium tuberculosis* (pathogen)	Increased infectivity upon surfactant deprivation induced by prolonged passages, which was rescued by exogenous surfactants	[13]
	Microfluidics	3D tubular bronchiole models in hydrogel + polymorphonuclear (PMN) leukocytes	*Aspergillus fumigatus* (fungus, pathogen)*,* *Pseudomonas aeruginosa* (pathogen)	A mutant with lower pathogenicity (*ΔlaeA* knockout *A. fumigatus*) induced higher cytokine secretions (IL-1β, IL-8) than the wild type The secretions of pro-inflammatory cytokines significantly higher when the epithelial cells were exposed to the volatile compounds from *A. fumigatus* and *Pseudomonas aeruginosa* at the same time than exposed separately	[102]
Urological	Well plate	Human bladder cancer cell line	Uropathological *E. coli* (UPEC, pathogen) + *Lactobacillus spp.* (pathogen)	Probiotics may prevent UPEC infection	[112]
	Porous membrane-assisted	Primary cells from bladder biopsy and derived transformed cells	*Enterococcus faecalis* (pathogen)	Apical urine facilitated differentiation of the bladder epithelial cells 2 h of infection formed IBCs	[113]
	Organoids	Murine bladder organoids + primary neutrophils	UPEC (pathogen)*,* microinjected	Locations of initial bacterial infection and regrowth after antibiotic treatment UPEC clearance by and escape from neutrophils	[114]
	Microfluidics	Human bladder epithelial cells + endothelial cells + neutrophils	UPEC (pathogen)	Increased infectivity by cyclic strains Transendothelial and transepithelial migration of neutrophils upon UPEC infection Categorized different subsets of IBCs and their regrowth patterns after antibiotic treatment	[42]

### Intestine

The human intestine is the primary habitat for commensal microbes in the human body, with approximately 98% of commensal bacteria colonizing the digestive tract, particularly the large intestine. As the evidence for the impact of commensal gut microbiota on overall health and intestinal homeostasis continues to accumulate, in vitro coculture of gut bacteria with host cells has attracted tremendous attention. However, the unique oxygen microenvironment of the intestine poses a significant challenge in developing a commensal gut bacteria coculture model. In healthy human subjects, the lumen of the intestine is deprived of oxygen, with 10–30 mmHg (1–4%) in the small intestine and 0.1–1 mmHg (0.01–0.2%) in the large intestine, while the intestinal tissue (serosa) is highly vascularized with measured oxygen tension of 80–100 mmHg [64]. Therefore, two conflicting oxygen environments should be generated within one system, an oxygen-deprived apical compartment for the gut bacteria and an oxygen-rich compartment for the human cells for proper functions and survival. Corresponding to the habitat environments, the gut bacteria of healthy adults are mostly anaerobes which are usually established typically before age 3 by substituting aerotolerant early colonizers [65]. In the small intestine, facultative anaerobes constitute the healthy microbiota, while 99.9% of commensal gut bacteria detected from human stool samples, which likely represent the luminal microbiota of the large intestine, are obligate anaerobic bacteria that cannot survive when exposed to oxygen [66]. The oxygen-deprived luminal environment of the colon where commensal gut bacteria can thrive play critical roles in protecting the intestine tissues from facultatively anaerobic enteric pathogens, such as *Salmonella*, *Yersinia*, *Shigella*, and enterohemorrhagic *Escherichia coli* (EHEC) in multiple ways. For example, strictly anaerobic commensal bacteria consume nutrients that enteric pathogens could use to limit their proliferation or produce chemicals that inhibit their virulence [67]. Also, depleting oxygen creates a hostile environment for enteric pathogens to express virulent factors [67]. Therefore, generating an oxygen gradient is critical for correctly reflecting the healthy intestinal microenvironment, successfully coculturing commensal bacteria with the host cells, and investigating infection of enteric pathogens in vitro. Recently, a few innovative in vitro intestinal model systems with physiological oxygen gradients have been reported and used for gut bacterial coculture, which will be discussed later in this section.

Given the heavy loads of commensal bacteria in the intestine, it is not surprising that many bacterial coculture studies have focused on gut bacteria. Earlier coculture studies before the advent of the “microbiome era” primarily focused on the bacteria that can be cultured in an aerobic environment. Bacterial coculture studies in an aerobic condition with gastrointestinal pathogens, such as *Salmonella*, *Yersinia*, or EHEC, showed the invasion mechanisms at cellular and molecular levels. Various probiotic strains that can survive in the oxygen-rich environment also have been cocultured with human intestinal or colon epithelial cells aerobically, sometimes with immune cells, and their protective roles were investigated in the context of chemically induced inflammation or pathogenic infections. For example, probiotic strains *Lactobacillus gasseri* 33323 and *E. coli Nissle* 1917 were cocultured in a 3D intestinal model generated by micromolding poly lactic-glycolic acid scaffold with and without pathogenic strains, *Salmonella typhimurium* 14038 and *Pseudomonas aeruginosa* 15692 [38]. This study showed that both probiotic strains improved the host cell survival against the pathogen infection, and each strain may have different spatial distribution in the crypt-villi axis. Immune components, such as neutrophils, macrophages, or monocytes, are often introduced to recapitulate the innate immune response in intestinal models [52, 53]. A recent study reported a triple culture system consisting of human epithelial cell lines, cell line-derived macrophages or monocytes, and bacteria, its use to model host–microbe–parasite interaction [68]. Using this model, the authors showed that the excretory/secretory products (ESPs) of the parasite *Teladorsagia circumcincta* mitigated the detrimental effect of *E. coli* on the human intestinal epithelium in the presence of the immune cells and altered the cytokine profiles.

HuMix model is one of the first intestinal models that mimicked the oxygen gradient of the in vivo human colon in a microfluidic device [69]. This microfluidic model consists of three channels separated by a nanoporous membrane and a micro-sized porous membrane. Bacteria are inoculated on the top channel separated by the nanoporous membrane from the human intestinal cells cultured on the microporous membrane. In this study, an anaerobe *Bacteroides caccae* was cocultured for 24 h with the human cells in combination with a facultative anaerobic bacteria *Lactobacillus rhamnosus GG* (*LGG)*, exploiting its oxygen consumption which presumably suppresses oxygenation of the anoxic medium. The authors found that coculturing *B. caccae* and *LGG* induced extensive transcriptional responses distinct from those observed when coculturing *LGG* alone. They also showed that the oxygen environment significantly altered the bacteria-driven gene expression patterns, urging the importance of incorporating the proper oxygen environment. One drawback of this model is that the microbes are physically separated from the human cells by a nanoporous membrane and are not allowed to bind to human cells, which can have significant effects. Nevertheless, the HuMix model is one of the first model systems that focused on the physiological oxygen environment of the colon and demonstrated its importance. Recently, the HuMix model has been used to screen cancer-related metabolites by coculturing *Fusobacterium nucleatum*, a colorectal cancer-related bacterium, with a patient-derived colorectal cancer cell line [70].

Emulate’s Organ chip is one of the best-recognized microfluidic model systems, as described earlier. Gut Chip [71], Intestine Chip [72], and Colon Chip [73] use the same microfluidic chip design with some differences in cell compositions. A physiological oxygen gradient was generated on the Intestine Chip that contained human primary ileal (the last part of the small intestine) epithelial cells and primary endothelial cells by flowing the deoxygenated medium in the apical channel and the oxygenated medium in the basolateral channel. This enabled coculturing apically inoculated *Bacteroides fragilis* and a complex community of gut bacteria collected from the human feces and then stably maintained in gnotobiotic mice. The peristaltic movement (one of the hallmarks of the Organ Chip system that will be discussed later) was not required for the coculture. Another group used a similar design of the microfluidic chip, named “anoxic–oxic interface (AOI) on-chip, to coculture anaerobic bacteria with the colon epithelial cell line without endothelial cells under an oxygen gradient [74]. *Bifidobacterium adolescentis* and *Anaerobutyricum hallii* (*A. hallii*) in this coculture with the oxygen gradient maintained high viability, although the bacteria proliferation is not tested. A “mesofluidic” culture platform, GuMI (Gut–Microbiome physiomimetic platform), is a dynamic culture system that can be used with cell culture inserts. It can generate the oxygen gradient in the commercial cell culture inserts (e.g., Transwell) using custom-made fluid control modules that enable flowing deoxygenated culture media [75]. These modules control the apical flow, which flows continuously, and the basolateral flow, which recirculates. A fastidious oxygen-sensitive bacteria, *Faecalibacterium prausnitzii* (*F. prausnitzii*), was successfully cocultured with the organoid-derived primary human colon epithelial cells for 96 h. The transcriptomic data indicated that cells exposed to apical and basolateral flows were more differentiated than those in static conditions. The oxygen gradient expectedly increased hypoxia-inducible factor 1-a (HIF-1) gene expression compared to a conventional aerobic condition and induced changes in the genes associated with HIF-1A. More importantly, coculturing a single strain of bacteria, *F. Prausnitzii*, extensively altered the gene expression patterns related to inflammation and Toll-like receptor (TLR) signaling, supporting the anti-inflammatory effect of *F. Prausnitzii*. Finally, a pumpless microfluidic model system was also developed using gravity-driven flow for coculturing gut bacteria with human colon epithelial cells in an oxygen gradient [76]. The oxygen gradient was established by placing the culture device in the anaerobic environment while flowing an oxygenated medium located in an aerobic condition through the tubing. With an optimized flow rate, *E. coli* were cocultured with human colon epithelial cell lines under this oxygen gradient without losing viability.

Physiological oxygen gradients were also generated in static intestinal coculture models [77, 78]. These static models can utilize cellular oxygen consumption to remove oxygen. In these models, a cap or plug is installed in the cell culture insert to block apical oxygen influx creating a semi-closed apical side. Then, cellular oxygen consumption effectively removes oxygen and maintains the anaerobic condition of the apical side without necessitating any anaerobic flow. Meanwhile, oxygen in the atmosphere dissolves in the basolateral medium and reaches the cells through the porous membrane. Implementing the oxygen gradient improved tolerance to *Clostridioides difficile* infection compared to an anaerobic condition, suggesting the importance of implementing a physiological oxygen gradient [77]. With this device, probiotic and commensal strains, including *LGG*, and *Bifidobacterium adolescentis (B. adolescentis)*, *A. hallii* were successfully cocultured with the primary human colon epithelial cells for 24 h [79]. This study showed a donor-invariant anti-inflammatory effect of a butyrate-producing bacteria *A. Hallii* while donor-dependent tolerance to *LGG*. A similar static intestinal model system with a physiological oxygen gradient, namely Intestinal Hemi-Anaerobic Coculture System (iHACS), was developed for gut bacterial coculture [78]. In this model, the cells were incubated in a completely anaerobic environment for 2 days first, then the anaerobic apical environment was preserved by installing a rubber stopper in the cell culture insert. Four bacterial species were individually cocultured with the primary human colon epithelial cells, and their influences on proliferation and differentiation marker gene expressions were assessed. In particular, *Akkermansia municiphila* grew in the coculture without exogenous nutrients, confirming its mucus-degrading capability. Finally, organoids were used for coculturing a single bacterial strain or a complex community of gut bacteria through microinjection [34, 35, 80], although it is not clear if the luminal side of the organoids is anaerobic enough to support obligate anaerobic gut bacteria. One study revealed that the oxygenation level of the organoid lumens is heterogeneous, ranging from 3 to 5% [81] and a possibility of the microbial composition being skewed by the oxygen tension higher than the in vivo lumen cannot be ruled out in the bacterial coculture in organoids.

In addition to the oxygen environment, several factors, including shear stress by content passaging, peristaltic movement (small intestine), and the presence or absence of the mucus layer, are important for determining the tolerance of the host cells against bacterial challenges. A significant trend in developing in vitro intestinal model systems is the integration of multiple host tissue components, such as immune cells, stromal cells, endothelial cells, and recently identified neurons. Introducing gut microbiota, an essential part of the gut ecosystem in vivo, into complex tissue models will provide more accurate and comprehensive model systems to understand the broad impact of the gut microbes and test platforms for pre-clinical studies.

### Skin

The skin forms one of the largest barriers that interact with microbes [82]. Human skin has three layers from outer to inner layers, the epidermis, dermis, and hypodermis, but phenotypes, such as thickness, compositions, microstructures, and biochemical requirements for differentiation, vary depending on the specific locations within the body. Various secretory glands and microstructures, including sweat, sebaceous, and apocrine glands, hair follicles, immune cells, nerves, and blood/lymphatic vessels, are present in the skin. In healthy conditions, considerable loads of microbes are constantly in contact with the skin, and even the buried microstructures of hair follicles and glands are colonized with microbes [82]. Compositions of skin microbes are site-specific, but most skin bacteria are aerobic.

Europe banned animal testing for cosmetic products in 2013 [83], which facilitated the development of in vitro human tissue models. Now, in vitro skin models have become a major testing platform for skin, with at least 13 commercially available [84]. Many skin models have a single layer of epidermis that consists of keratinocytes or a double layer that is comprised of the epidermis with keratinocytes and dermis with fibroblast in collagen. These models adopt an air–liquid interface (ALI) culture reflecting the environment that the skin in vivo is exposed. Well-established host tissue models and undemanding collection and maintenance of the skin bacteria enabled the achievement of a long-term bacterial coculture. For example, a long-term (8 days) microbial–skin tissue coculture model was established using a commercial full-thickness 3D skin model, with two skin bacteria isolated from healthy skins, *Micrococcus luteus* (*M. luteus*) and *Pseudomonas oleovorans* (*P. oleovorans*), separately and together in an ALI [85]. These commensal bacteria altered secretions of cytokines, antimicrobial peptides, and growth factors of the human cells in a species-dependent manner. When two bacteria were cocultured simultaneously with the skin tissue, the influence of *P. oleovorans* generally dominated over *M. luteus*. Another study reported independent cocultures of eight different bacterial strains and a mixed community with a commercial skin tissue model consisting of epidermal and dermal layers [86]. Transcriptomic analysis after 18 h coculture revealed that the transcriptional changes by a mixed community are distinct from those induced by individual bacterial treatment. Also, changes in the skin tissue phenotypes, such as a decreased thickness of the nucleated epidermal layer and proliferative cell populations, were the most prominent after 5 days of bacterial treatment in the samples treated with the mixed community. Moreover, significant increases in gene expression of loricrin and filaggrin which are important in skin barrier structure and functions were detected only in the mixed community treated samples, illustrating the importance and necessity of introducing a bacterial community rather than a single strain in an in vitro study to reflect the in vivo skin. These studies demonstrate that skin commensal bacteria influence and modulate cell fates, barrier functions, and immune responses of the skin tissue.

Pathogenic infections have also been studied using in vitro skin tissue models. An infection model for melioidosis was developed using a double-layer model consisting of keratinocytes over fibroblast embedded in fibrin hydrogel [87]. Melioidosis, also known as Whitmore’s disease, is a potentially lethal infectious disease caused by *Burkholderia pseudomallei* (commonly known as *Pseudomonas pseudomallei*) prevailing in tropic regions. With a surrogate model microbe *Burkholderia thailandensis*, the study showed that the infection slowed wound healing, triggered inflammasome, and induced ejection of the keratinocytes.

While in vitro model systems based on the skin cell culture have been useful in investigating the consequences of infection with a known pathogen, it requires days to grow the cells, which may not be appropriate for the fast identification of pathogens and therapeutic strategies in clinical settings. An ex vivo model from a biopsy of the infection site that does not require cell cultures for days can be more efficient in clinical settings. An ex vivo model system with full-thickness skin explant was developed to investigate neutrophil migration to pathogen-infected tissue [88]. Full-thickness skin explant samples with all three layers of skin were collected using a triple-edged microbiopsy needle and subjected to *Staphylococcus aureus* infection. Then, the infected tissue was placed in a channel of a microfluidic system. Another channel in the device isolated neutrophils from autologous whole blood in situ. The isolated neutrophils migrated toward the infected tissues, not toward the uninfected tissue, and antibiotic treatment significantly reduced neutrophil migration. This model can be used for diagnostics and screenings for appropriate antibiotics using biopsy samples and autologous blood samples of patients.

Degradation and contraction are the main drawbacks of the collagen-based skin model, which synthetic polymers can minimize [89]. Also, current in vitro skin models used for bacterial cocultures mostly lack three-dimensional microstructures and associated functions such as glands or follicles. Recently bioengineering approaches achieved three-dimensional microstructures of skins [90]. It will be interesting to see how skin microbes are associated with microstructures or gland functions in vitro. More information on the current development of skin models can be found in recent reviews [84, 91].

### Female Reproductive Tract (FRT)

The FRT is composed of the vagina, cervix, uterus (endometrium), fallopian tubes, and ovaries. Like other tracts in the human body, FRT exhibits physiological, immunological, and microbial gradients. The epithelial organization also varies throughout the tract, with stratified squamous epithelium present in the lower genital tract from the vagina up to the ectocervix, transitioning at the transformation zone to the simple columnar epithelium that lines the upper FRT [92]. The epithelium is protected by cervicovaginal mucus which contains antimicrobial peptides and other immunologically active proteins. Oxygen concentration within the FRT decreases from the fallopian tube at 5–7% to 2% in the uterus [93]. The microbiome of the healthy FRT commensal microbiomes are relatively less diverse than other organs, primarily composed of *Lactobacillus* strains which are lactic acid-producing facultative anaerobes. The acidic environment created and maintained by the commensal *Lactobacillus* trains in the FRT contributes to protecting the tissue from other microbial challenges. Commensal bacterial loads in the lower FRT are 10^2^–10^4^-fold greater than in the upper FRT. Similar to other organs, commensal bacteria in the FRT have an impact on the barrier functions of the epithelium and tissue homeostasis [92]. Bacterial vaginosis (BV) is a common vaginal condition characterized by dysbiosis of vaginal microbes with a decrease of *Lactobacillus spp.*, an increase in pH, and an increase in anaerobic or other bacterial vaginosis-associated bacteria (BVAB). BV affects about 30% of childbearing-age women in the United States and is one of the major concerns in female reproductive health [94].

The well-defined and relatively less diverse compositions of the commensal and BV-associated microbiota in the FRT than other organs have facilitated the development of various bacterial coculture models. Immortalized cells or primary cells in the conventional submerged culture or an ALI, including commercial models with primary cells (e.g., EpiVagina, VEC-100), have been cocultured with the commensal *Lactobacillus* strains or BVAB strains, or both. In one study, the immortalized epithelial cell lines from different regions of the FRT were cultured aerobically on Transwell, and then, commensal or BV-associated bacteria were cocultured in an ALI for 24 h [95]. Endocervical epithelial cells, located in the upper female genital tract in vivo, showed significantly more sensitive and robust immune responses to the bacteria than ectocervical or vaginal epithelial cells, which is inversely correlated to the bacterial loads in vivo. Also, BV-associated bacteria and one of *Lactobacillus* species, *Lactobacillus vaginalis,* increased secretions of pro-inflammatory cytokine IL-8, Gro-alpha (CXCL1), and antimicrobial peptide hBD2 than the commensal bacteria did, which is consistent with the cytokine readings from the clinical samples.

The efficacy of microbicidal compounds was tested using an in vitro coculture model. A simple monolayer coculture model with immortalized cells on well plates was utilized to determine the effectiveness of the microbicidal compound cellulose sulfate with and without vaginal microbes [96]. First, without cellulose sulfate, 5 days of anaerobic coculture with the four different individual bacterial strains revealed that BV-associated bacteria *Prevotella bivia* and *Atopobium vaginae* (*A. vaginae*) induced higher secretion of a pro-inflammatory cytokine IL-8 and NF-kB activity than commensal strains, *Lactobacillus crispatus* (*L. crispatus*)*,* and *Lactobacillus Acidophilus*, consistent with the other study. Moreover, cellulose sulfate did not significantly alter the epithelial cell viability without bacteria, but when bacteria were present, it increased the expression of innate immunity-related proteins, such as IL-8 and secretory leukocyte protease inhibitors in a dose-dependent manner, illustrating the necessity of including proper microbiota in a pre-clinical model.

Meanwhile, a three-dimensional vaginal cell aggregate model was generated by culturing the immortalized primary cells in a rotating wall vessel reactor with collagen-coated dextran beads [97]. The cell aggregates were cocultured with four individual bacterial strains (two commensals *Lactobacillus iners* and *L. crispatus* and two BV-related *P. bivia* and *A. vaginae*) anaerobically in well plates for 24 h. The cells responded to the bacterial challenges in a species-dependent manner. *A. vaginae*, a BV-associated bacterial strain, tended to increase the gene expression of some pro-inflammatory cytokines, antimicrobial peptide defensins, and protein secretions of cytokines, including IL-6, IL-8, TNF-α, and IL-1β, which is consistent with the results with the other platforms.

Recently, a vagina-on-a-chip model adopting the Organ Chip from Emulate was used for bacterial coculture [98]. Primary vaginal epithelial cells, and uterine fibroblast cells were seeded on each side of the PDMS porous membrane. Continuous perfusion in the bottom channel and intermittent flow in the top channel yielded multilayered, squamous, and stratified epithelial cell layers with correct differentiation markers. The epithelial cells responded to a female sex hormone β-estradiol, by altering gene expression levels of specific targets, including estrogen receptor 1 (ESR1), progesterone receptor (PGR), and claudin 17 (CLDN17). This model recapitulated the physiological pH (around 4.7) and bacteria-derived lactate production by coculturing a strain or multi-strain consortia of *L*. *crispatus* for 4 days. Commensal bacterial coculture suppressed pro-inflammatory cytokine secretions, including IL-8, IL-6, IL-1a, IL-1β, and interferon-γ inducible protein (IP-10). When BV-associated bacteria were cocultured, the pH significantly increased to 5.1, no lactate was detected, and the pro-inflammatory cytokines increased compared to the control without bacterial exposure, which is consistent with the previous studies.

In vitro coculture models have successfully recapitulated the expected immune responses by commensal and BV-related strains. Still, some critical aspects of FRT physiology are yet to be recapitulated, such as mucus production, oxygen environment, hormonal changes, or menstrual cycles, which are expected to impact host–microbial interactions.

### Respiratory Tract

The respiratory tract, exposed to particles, chemicals, and microbes, is one of the most susceptible organs to biological and chemical challenges. Respiratory infections are one of the leading causes of death for adults and children, with an estimated 2.4 million death annually due to lower respiratory infections, even before the COVID-19 pandemic [99]. The upper (nasal and oral cavities, larynx, pharynx, and throat) and the lower (trachea and lung) respiratory tracts have distinct physiological and biological characteristics. In a healthy body, microbial gradients exist along the respiratory tract, with the highest bacterial biomass in the upper respiratory tract decreasing downward [100]. Studies have shown that germ-free animals have impaired lung development, such as smaller size and less mature alveoli, indicating that respiratory commensals have significant impacts on the respiratory development and health of the host, similar to the intestine [100]. Also, it is suggested that commensal microbes in the upper respiratory tract protect the lungs from opportunistic pathogen overgrowth and dissemination [100].

Host cells in many in vitro respiratory model systems are cultured in an ALI, reflecting the physiological environment that faces gas. In lung models, an ALI culture increased the epithelial secretion of surfactants which prevents dehydration by stabilizing the thin liquid layer in vitro [41]. Also, airway epithelial cell lines (e.g., Calu-3) cultured in an ALI produce mucus, improving the host cell viability by forming a protective barrier for the host cells [101]. In this condition, the Calu-3 cells were successfully cocultured with a nasal bacterial community and a probiotic strain, *Lactobacillus sakei*, independently for 3 days without loss of the host cell viability. Interestingly, the addition of THP-1 macrophage-like cells altered the microbiome compositions and increased the phenotypic diversity of the bacterial community.

Dynamic features of the respiratory organs, such as cyclic contraction and release of breathing motion and blood flow, were recapitulated using bioengineered systems. A microfluidic-based lung-on-a-chip model was developed to simulate cyclic movement, which conventional static models cannot replicate. The model utilizes two pneumatic side channels connected to each side of the main channels [41]. Applying negative pressure inside the two side channels contracts the side channels. This contraction pulls the walls of the main channels on both sides and stretches the stretchable porous membrane where the cells are grown in the middle of the main channels. This innovative design was also used to mimic the peristaltic movement of the intestine and the stretching of the bladder [42, 71]. The lung-on-a-chip model recapitulated the transendothelial and transepithelial migration of the neutrophils and the phagocytosis of GFP-*E. coli* under inflammatory stimulation with TNF-α [41]. Interestingly, the cyclic strains increased the production of reactive oxygen species in response to silica nanoparticles and nanoparticles in general, highlighting the importance of incorporating proper movement for a lung model and suggesting a possible underestimation of nanotoxicity in static models. The effect of native surfactant secretion of the host cells on the *Mycobacterium tuberculosis* infection, the cause of tuberculosis, was investigated using the same device [13]. Prolonged passages of the primary alveolar epithelial cells resulted in surfactant deficiency, which significantly increased the intracellular infectivity in the epithelium (cultured in an ALI) and the macrophages. Adding exogenous surfactants effectively attenuated the infection by inhibiting bacterial adhesion.

A three-dimensional tubular bronchiole model comprised of an airway channel and vascular channels embedded in collagen/fibrinogen hydrogel was developed to model fungal infection [102]. The spores of a fungal pathogen, *Aspergillus fumigatus* (*A. fumigatus*), inoculated into the lumen of the bronchial epithelial channel formed fungal filaments sprouting through the epithelium. Upon the fungal infection, polymorphonuclear (PMN) leukocytes added into microvascular channels migrated through the endothelial cells, the hydrogel, and toward the fungal filaments. A mutant with lower pathogenicity (*ΔlaeA* knockout *A. fumigatus*) induced higher cytokine secretions (IL-1β, IL-8) than the wild type, suggesting that the early immune responses have a role in clearing out the pathogens. The authors also showed that the secretions of pro-inflammatory cytokines were significantly higher when the epithelial cells were exposed to the volatile compounds from *A. fumigatus* and *Pseudomonas aeruginosa* at the same time than exposed separately.

Various respiratory model systems are reviewed elsewhere [103]. However, most studies have neglected the potential impacts of respiratory microbes. Recent studies suggest that commensal bacteria may have significant roles in protecting against pathogenic infections. For example, in the mice models, commensal bacteria have been shown to inhibit the colonization of lung pathogens such as *Pneumococcus* [104, 105]. Some respiratory infections are caused by opportunistic pathogens that are normal members of healthy commensal microbes and only become pathogenic when dysbiosis occurs, such as prolonged hospitalization or intubation. Commensal bacteria coculture models may help more accurately understand how normal commensals become pathogenic and how epithelial cells respond to pathogen infection and other challenges, such as micro/nanoparticle inhalation.

### Urinary Tract

The urinary tract is the drain system of the body. It consists of the upper tracts, including kidneys and ureters, and lower tracts, with bladder and urethras. The kidney is a complex organ responsible for filtering out wastes, regulating electrolyte concentrations and acid–base balance of the blood, and secreting hormones to control blood pressure and composition. The nephron, a functional kidney unit, contains at least 16 different epithelial cell types with various transport proteins in the nephron. Reproducing all the fully matured cell types of the kidney in vitro remains a significant challenge. To date, success has yet to be reported in the in vitro maturation of kidney epithelium into all different types. The urinary tract from the renal pelvis, where the urine is funneled to the ureter, down to the urethra, has a special lining of cells, urothelium, which is an epithelium with 3–7 layers of cells, including basal, intermediate, and umbrella layers found from the basal to luminal sides [106].

For many years, the urinary tract was believed to be sterile as conventional bacterial culture methods did not detect bacteria in the urines of healthy, non-infected subjects. Urinary bacteria were associated with renal infections. However, recent research using different sample collection methods (e.g., catheterization, subpubic punctures), genomic sequencing, and bacterial cultures under different oxygen conditions have revealed that the urinary tract does have its own unique microbiota, with some overlap with the gut and genital microbiota, at 10^3^–10^5^ bacteria/mL urine of healthy adults [107–111]. Despite these findings, our understanding of the urinary microbiota is still relatively limited, and further research is needed to fully understand the roles and implications of these commensal urinary microbes in renal health and disease.

On the other hand, urinary tract infection (UTI) is comparatively better defined. About 80% of UTIs are caused by uropathological *E. coli* (UPEC). The high recurrence rate (~ 25%) of this infection is at least in part explained by the presence of intracellular bacterial communities (IBC). These biofilm-like bacteria proliferate inside the host epithelial cells and provide the ability to escape from innate immunity and antibiotic treatment. UPEC can penetrate even deeper, forming quiescent intracellular reservoirs (QIRs) that may reinfect the host even after antibiotic treatment.

In vitro bacterial coculture studies for the urinary tract have primarily focused on UTIs. One such study utilized a simple model of a human bladder cancer cell line to demonstrate the inhibitory effect of a probiotic strain (*Lactobacillus spp*.) on the growth of a UPEC strain, suggesting that probiotic strains may be an effective method for preventing UPEC infections [112]. Also, primary cells from bladder biopsy have also been used for modeling the UPEC infection. Transwell culture of primary cells from human bladder biopsy and their derivative transformed cells expressed key markers of the human urothelium, including cytokeratin (CK) proteins, uroplakins-III, and glycosaminoglycan upon differentiation which required apical urine [113]. The study found that 2 h of infection with a uropathogenic bacteria *Enterococcus faecalis* was enough for the bacteria to get into the host cells and form IBCs in vitro.

Impressive details of the UPEC were reported using high-resolution live cell imaging on the murine bladder organoids with microinjected UPEC [114]. The authors showed that the initial bacterial growth after inoculation was observed in the lumen of the organoids and, on the contrary, regrowth after antibiotic washout occurred exclusively in the wall of the organoids. The primary neutrophils embedded in collagen gel with the organoids migrated toward the lumen of the UPEC-infected organoid cultures and cleared out the bacteria but not toward the uninfected organoids. Moreover, the UPECs in the bladder walls escaped from neutrophil clearance. Through high-resolution three-dimensional scanning electron microscopic imaging, the authors categorized the UPEC by their locations within the organoids and showed that the bacteria in the lumen and IBC displayed distinct morphologies.

A bladder chip using the Organ Chip from Emulate with the human bladder epithelial and endothelial cells was used to model UPEC infection [42]. By exploiting the cyclic stretching capability of the Organ Chip, the authors were able to replicate the strain on the in vivo bladder during filling and emptying. The study found that this cyclic strain increased the degree of UPEC infection. When UPEC was inoculated for 2 h in the apical (top) channel, and the neutrophils were introduced into the basolateral (bottom) channel, neutrophils transmigrated through the endothelial and subsequently, the epithelial cell layers and formed neutrophil extracellular traps (NET), a defense mechanism of neutrophils in addition to engulfing the bacteria. The authors observed different types of IBC subsets and their regrowth dynamics before and after serial antibiotic treatments.

**Fig. 2:**
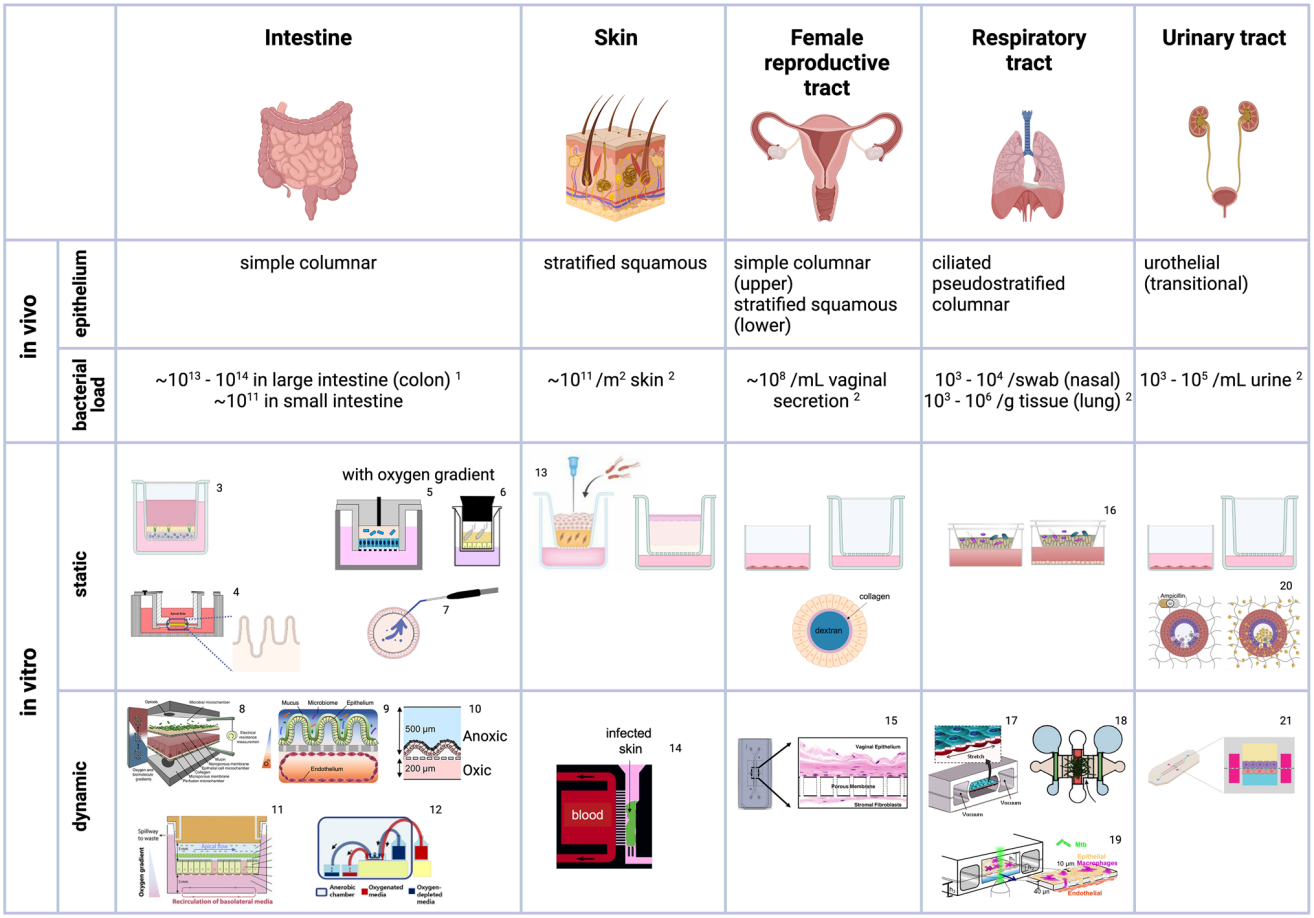
Human organs with significant bacterial loads and noteworthy examples of organotypic microphysiological systems with bacterial cocultures described in Sect. 4. ^1,2^ Bacterial loads in the human adult intestines and other organs are referenced from [20] and [111], respectively. ^3–21^ Images are reproduced, respectively, from [13, 34, 41, 42, 68, 69, 72, 74–76, 78, 79, 87, 88, 98, 101, 102, 114, 116] with the permission of publishers. The image was created with BioRender.com

It will be interesting to assess the roles of urinary commensal bacteria in the development and differentiation of uroepithelial cells, the homeostasis of the urinary tract, and UTIs in vitro. More data on the commensal bacterial community from clinical samples will facilitate the in vitro coculture studies. It may be necessary to create the oxygen gradient for the coculture studies, since a significant proportion (35 ~ 39%) of the urinary microbiota seem to be anaerobic bacteria that are overlapped with the gut or vaginal commensal bacteria [115] (Fig. 2).

## Challenges and Opportunities in Organotypic Bacterial Cocultures

Although the human cells in a healthy body manage to coexist with commensal bacteria, maintaining the high viability of both host epithelial cells and commensal bacteria in vitro is unexpectedly challenging. Host cell damage is somewhat expected in pathogenic bacterial coculture studies, but the commensal or probiotic strain-induced impairment of host cell viability also frequently occurs. One of the main causes of host cell death in bacterial cocultures is bacterial overgrowth, especially in static submerge culture, leading to the deprivation of nutrients and accumulation of excess toxic substances (endotoxins) for host cells. Strategies to successfully maintain bacterial coculture can be drawn from the in vivo environment, where commensal bacteria typically face suboptimal conditions, such as continuous or periodic washout and limited nutrients. Modifying culture medium compositions (with less nutrients or less efficient nutrient sources than optimal) and lowering bacterial inoculation or multiplicity of infection may alleviate host cell damage. A continuous or periodic flow in the luminal compartment can significantly improve coculture capability by washing out excessive bacteria and associated toxic compounds while providing a fresh medium. A reduced dose of an antibiotic has been used to control bacterial growth, but it is hardly reliable and carries the risk of developing an antibiotic-resistant strain.

Another common issue in bacterial coculture is the lack of important phenotypes in the host cells that are directly associated with antibacterial defense mechanisms. To be better equipped to manage commensal bacteria and protect from pathogens, an in vitro system needs to replicate more closely and accurately the in vivo tissues that coexist with hundreds of trillion microbes. The mucus layer is one of the vital protective mechanisms often missing in the in vitro mucosal epithelium (such as oral, nasal, intestinal, and vaginal epithelium). The mucus layer physically separates bacteria from the host cells and contains antimicrobial substances. Therefore, the presence or absence of the mucus layer significantly impacts the host cell viability, as well as bacterial (over)growth in vivo and in vitro. However, only a few cell lines can secret the mucus in a way that forms an adequate to separate bacteria from the epithelium. Alternatively, mucus from an external source (e.g., porcine stomach mucus) is often introduced to the model systems dissolved in the medium, but it rarely forms a layer that can separate bacteria from the underlying epithelium. Using primary epithelial cells producing more in vivo-like mucus layers when differentiated appropriately may provide higher tolerance to commensal and pathogenic bacteria better than some cell lines without mucus-secreting capability. Incorporating immune and stromal cells into the tissue models, as the in vivo tissues, may provide additional defense and support systems for epithelial cells, possibly with some design changes on biomaterials or culture medium compositions.

To better mimic in vivo tissue, the transport of biochemicals such as oxygen and nutrient in an in vitro model should be improved. Many current static models are limited by poor oxygen or nutrient delivery, leading to failure in impaired differentiation, long-term culture, and even cell death, which is directly related to bacterial coculture capability. Perfusion systems, such as microfluidic devices, have shown more in vivo-like cell behaviors, at least in part through improved oxygen and biochemical delivery. Similarly, dynamic batch culture systems with stirring significantly improved cell differentiations, as well as cell viability and culture periods, in brain organoid cultures, for example [117]. Introducing vasculatures is expected to significantly enhance the transport of biochemicals, including oxygen and nutrients. Additionally, it can offer a tool for observing whether or how microbes interact with endothelial cells and enter circulation in addition to epithelial–endothelial interactions.

Ultimately, to fully understand the local and systemic effects of host–microbial interactions, the complexity of the human organ tissues should be considered. Host epithelia are crucial components in studying microbial interaction with the host, but they are not the only host cell types that participated in host–microbial interactions. Human tissues consist of various cells, including stromal, muscle, vascular, neuronal, tissue-resident, and circulating immune cells, forming unique microstructures and microenvironments associated with the tissue functions. Non-epithelial cells may be impacted by human microbes directly or indirectly, affecting their behaviors, spatiotemporal distributions, microenvironments, and functions. Human tissue models with multiple cell types will be necessary to investigate the broad impact of host–microbial interactions in depth. In addition, organ-specific movements are also important factors of the host tissues that need further integration. Organ-specific movements, such as peristalsis, periodic stretching and contractions, and shear stress by fluid, can influence bacterial adhesion and growth as well as regulate host cell behaviors that involve defense mechanisms. Various microphysiological systems with dynamic stimuli and movements can be particularly useful for generating dynamic bacterial coculture platforms [118–120].

To date, most bacterial coculture studies have used a single strain of bacteria in the cocultures with host cells. This provides valuable knowledge of specific interactions between the bacteria and the host cells, for example, whether physical contact is necessary or whether bacteria-derived compounds can induce any effect without direct contact. However, to replicate the in vivo environment more accurately, a coculture system should include a community of commensal bacteria rather than one strain. As discussed previously, some studies have already introduced a community of bacteria, particularly for target organs with relatively simple and well-characterized microbiomes. These studies have found that communities of commensal bacteria can induce different or more intense responses from the host than one commensal strain and that interactions among the bacteria, including pathogen–commensal interactions, play significant roles in pathogen infection or maintaining tissue homeostasis.

Moving forward, it is crucial to thoroughly examine and validate the model system to ensure that the coculture conditions do not artificially alter the events occurring, particularly for organ model systems with complex microbiota, such as intestines. For example, whether and how the nutrients, the oxygen environment, or other components in the coculture artificially promote certain bacterial strains over other strains should be examined. One practical solution is to use a pre-defined model bacterial community with selected bacteria for validating the coculture ability of an in vitro model system. This allows for quantitative analyses that are more manageable and less costly than a donor-derived bacterial community. For the gut microbiota, synthetic bacterial communities with varying levels of complexity have been proposed [121–123], which can be useful for in vitro and animal model studies. Potentially, bacterial coculture methods can be standardized for drug development with specified microbiota and media compositions. In the future, other microbes that constitute the human commensal community may be included once more data are accumulated. Ultimately, coculturing donor-derived microbial communities with autologous host cells can pave the way for developing a personalized test platform for individualized therapeutic interventions or disease prevention.

Multi-organ model systems, such as body-on-chip or human-on-chip, can be enhanced by incorporating bacterial coculture to resemble the body more closely. The gut–organ axis, which highlights the connections between gut microbial compositions and the health of organs beyond the intestine, including the brain, liver, skin, lung, kidney, heart, and bone, can be studied using these systems. Multi-organ model systems can provide valuable platforms for investigating host–microbial interactions across multiple organs with greater control than animal studies. The pharmaceutical industry can benefit from these multi-organ model systems with bacterial coculture capability by studying the effect of gut microbes on the chemistry, metabolism, and absorption of orally administered medicines. Preclinical models with human cells and gut microbial coculture capability can reveal the microbe-derived metabolic and chemical changes and their effects on the absorption of drug candidates in human cells, improving the efficiency of orally administered drug development.

Long-term coculture studies have the potential to offer valuable insight into homeostasis and dysbiosis-associated spatiotemporal distributions of microbiota. In healthy individuals, the composition of the microbiome can vary significantly across different body sites and can change at different rates depending on the individual [124]. Some studies suggest that the rate of change itself could indicate the host's health status [125]. Currently, many coculture studies are limited to a few days at maximum (except for skin model systems), which may be not long enough time scale to study tissue or immune cell developments, chronic conditions, or disease pathogenesis such as cancer. By studying the influence of microbes on various biological and pathological events through long-term coculture, a deeper understanding of the complex interactions between the host and its microbiome can be achieved.

## Summary

Organotypic in vitro model systems have significantly advanced our understanding of the target organs in various ways and are likely to facilitate more efficient screening and testing for therapeutic interventions and disease prevention. With growing evidence on the influences of microbiota on human health, introducing commensal microbes is a logical next step to generate a more accurate and comprehensive in vitro model system for mechanistic studies or screening strategies of medical interventions, such as drug or dietary compounds. However, introducing bacterial species into an organotypic model system comes with new challenges and requires a thorough understanding of both host and bacterial cell behaviors for successful integration, troubleshooting, and analyses. More in vivo-like organotypic model systems achieved by culturing primary cells and adding immune components can capture important phenotypes critical for defense mechanisms and provide a comprehensive understanding of how human cells interact with and respond to microbes. Bioengineering approaches can add physiological features that conventional static model systems cannot, such as an oxygen environment, 3D microstructures, and mechanical stresses from cyclic strains, peristalsis, and fluid flow. By combining innovative bioengineering approaches with advanced microbiological knowledge and technologies, organotypic in vitro coculture systems with a complex microbiota community will offer valuable tools for understanding human health and exploring new therapeutic targets and preventive strategies.

## Data Availability

Data availability is not applicable to this article as no new data were created or analyzed in this study.
